# Deep learning model for automatic segmentation of lungs and pulmonary metastasis in small animal MR images

**DOI:** 10.3389/fbinf.2022.999700

**Published:** 2022-10-12

**Authors:** Edgar Lefevre, Emmanuel Bouilhol, Antoine Chauvière, Wilfried Souleyreau, Marie-Alix Derieppe, Aurélien J. Trotier, Sylvain Miraux, Andreas Bikfalvi, Emeline J. Ribot, Macha Nikolski

**Affiliations:** ^1^ Bordeaux Bioinformatics Center, University of Bordeaux, Bordeaux, France; ^2^ IBGC, CNRS, University of Bordeaux, Bordeaux, France; ^3^ BRIC, INSERM, U1312, University of Bordeaux, Pessac, France; ^4^ Service Commun des Animaleries, University of Bordeaux, Bordeaux, France; ^5^ Centre de Résonance Magnétique des Systèmes Biologiques, CNRS, University of Bordeaux, Bordeaux, France

**Keywords:** small animal MRI, motion-correction, pulmonary metastases, deep learning, automatic segmentation

## Abstract

Lungs are the most frequent site of metastases growth. The amount and size of pulmonary metastases acquired from MRI imaging data are the important criteria to assess the efficacy of new drugs in preclinical models. While efficient solutions both for MR imaging and the downstream automatic segmentation have been proposed for human patients, both MRI lung imaging and segmentation in preclinical animal models remains challenging due to the physiological motion (respiratory and cardiac movements), to the low amount of protons in this organ and to the particular challenge of precise segmentation of metastases. As a consequence post-mortem analysis is currently required to obtain information on metastatic volume. In this work, we have developed a complete methodological pipeline for automated analysis of lungs and metastases in mice, consisting of an MR sequence for image acquisition and a deep learning method for automatic segmentation of both lungs and metastases. On one hand, we optimized an MR sequence for mouse lung imaging with high contrast for high detection sensitivity. On the other hand we developed DeepMeta, a multiclass U-Net 3+ deep learning model to automatically segment the images. To assess if the proposed deep learning pipeline is able to provide an accurate segmentation of both lungs and pulmonary metastases, we have longitudinally imaged mice with fast- and slow-growing metastasis. Fifty-five balb/c mice were injected with two different derivatives of renal carcinoma cells. Mice were imaged with a SG-bSSFP (self-gated balanced steady state free precession) sequence at different time points after the injection of cancer cells. Both lung and metastases segmentations were manually performed by experts. DeepMeta was trained to perform lung and metastases segmentation based on the resulting ground truth annotations. Volumes of lungs and of pulmonary metastases as well as the number of metastases per mouse were measured on a separate test dataset of MR images. Thanks to the SG method, the 3D bSSFP images of lungs were artifact-free, enabling the downstream detection and serial follow-up of metastases. Moreover, both lungs and metastases segmentation was accurately performed by DeepMeta as soon as they reached the volume of $∼0.02\;mm^{3}$ . Thus we were able to distinguish two groups of mice in terms of number and volume of pulmonary metastases as well as in terms of the slow *versus* fast patterns of growth of metastases. We have shown that our methodology combining SG-bSSFP with deep learning, enables processing of the whole animal lungs and is thus a viable alternative to histology alone.

## 1 Introduction

There is a plethora of preclinical studies investigating the efficacy of innovative treatments on primary tumors as well as on the subsequent metastases, especially pulmonary ones. Indeed, metastases in lungs are of particular interest since they are a sanctuary of many cancer cells (Leong et al., 2006) probably due to the high oxygenated environment and the dense capillary network. Moreover, there is evidence that the number (Cho et al., 2015) and size (Javed et al., 2014) of the pulmonary metastases are related to the survival prognosis. To obtain such quantitative information, most preclinical studies perform post-mortem lung extraction to determine the amount of metastases and their average area or volume. Histology is frequently used due to its high sensitivity of detection, however this technique only allows the analysis of a few lung slices (Shimada et al., 2018; Pein et al., 2020). The number of metastases per slice and their average size (*mm* ^2^) are then measured and interpreted as markers of a treatment efficiency.

The usual imaging technique for detecting lung tumors is through a CT scan. However, the X-rays doses limit patient’s follow-up, repeated and close sessions, preventing early detection of the disease and patient monitoring during treatment. For example, the French Nuclear Safety Authority (ASN) has published an action plan (n°2011-DL-0019, 2011) aiming to limit the doses delivered to patients, and to favor examinations by Magnetic Resonance Imaging (MRI). MRI is a non-invasive and non-traumatic technique used to perform serial follow-ups to detect lesions. It is a method of choice in oncology since it does not involve ionizing radiation, thus enabling repeated and close sessions to monitor tumor growth. In small animal models, preclinical MRI makes it possible to assess the efficiency of cancer treatments before translation to human studies. However, lung imaging is challenging due to physiological motion (respiratory and cardiac movements) and to the low amount of protons in this organ. We intended to exploit this latter property to efficiently detect pulmonary metastases. For this purpose, the balanced Steady State Free Precession (bSSFP) sequence was chosen as it has been previously shown that high tumor contrast can be obtained in the brain and in the liver (Miraux et al., 2008; Ribot et al., 2011, 2015). When combined with the Self-Gating (SG) method, motion-induced echoes can be canceled, resulting in images of the abdomen without motion artifacts (Ribot et al., 2015). This sequence is also of high interest due to its high SNR and short scan time to obtain 3D stacks necessary to cover a whole organ with high spatial resolution. Nevertheless, to our knowledge, it has never been applied to detect cancer lesions in lungs.

Deep learning associated with MRI has gained a lot of interest in recent years for different image quantification tasks. Applications range from image acquisition and image retrieval to segmentation and disease prediction (Lundervold and Lundervold, 2019). This is particularly true for human brain diagnostics. For example, recent studies have been conducted to automatically segment tumors or metastases in the human brain, using deep learning, such as the DeepMedic Neural Network (Liu et al., 2017; Charron et al., 2018; Grøvik et al., 2020). In the case of segmentation of metastases within lungs of human patients, (Wang et al., 2019), have proposed a deep learning patient-specific method to segment metastases in expert annotated VOIs around them. The model is trained specifically for each patient on early timepoints and can be used in follow-up MRI scans of the same patient, thus lacking the generalization capacity.

However, to date few studies are conducted on small animals such as rats or mice (see e.g. for (Tan et al., 2018) the segmentation of the left ventrical) and, up to our knowledge, no studies has been conducted on lungs, as a result of very low standardization of preclinical protocols, of large variety of mouse lineages, tumor models, as well as of MR sequences and MR instruments (mainly reception coils). Moreover, the low number of animals used in preclinical studies in order to comply as much as possible with the 3R regulations (Replace, Refine, Reduce) guidelines for animal experimentation, represents an additional challenge for downstream automation of image quantification.

In the case of cancer-related studies, the lack of methods for tumor detection and segmentation specific for small-animal preclinical studies, results in manual and time-consuming segmentation by experts. In the case of lung tumors, the development and validation of automated solutions is further hampered by the fact that there are no public databases of mouse or rat MR lung images. Even for human lung images, to our knowledge, there are only two public databases (Lung Image Database Consortium image collection LIDC-IDRI and ELCAP Public Lung Image Database), both based on CT scans, which limits the development of AI approaches.

In this study we developed a complete methodological pipeline for the automatised analysis of lung and metastases in mice, consisting of an MR sequence for imaging acquisition and a deep learning method for lung and metastases segmentation. This deep learning method enables the measurement of metastases volume in lungs at a given time and the assessment of their longitudinal growth. To ensure reproducibility and foster the use of our method by a large scientific community, all relevant software resources and MR image data are made publicly available. As such, our full pipeline consisting of an MR sequence and downstream automatic image quantification with DeepMeta, constitutes the first step toward standardization of lung and metastases imaging and segmentation in mice, while our MR image database constitutes the first publicly available data resource to foster further methods development.

## 2 Materials and methods

### 2.1 Animals

The murine renal cancer RENCA cell lines were maintained in RPMI-1640 (Eurobio) supplemented with 10% (v/v) FBS and 1% (v/v) penicillin-streptomycin, and incubated at 37°C with 5% CO2. To acquire time-series images of metastases growth showing different growth patterns, 2 cell lines were used: (i) interleukin-34 (il34) knock-out by Crispr/Cas9 method using the 5′-GAC​CTT​ACA​GGC​TAC​CTT​CGG​GG-3′ targeted sequence for slow-growing metastases pattern and (ii) targeting the LacZ gene using the 5′-TGC​GAA​TAC​GCC​CAC​GCG​AT-3′ targeted sequence for fast-growing metastases pattern. These 2 cell lines were further injected either intravenously or orthotopically under the renal capsule. For sub-capsular implantations, 1 × 105 RENCA cells were injected under the left kidney capsule of 6–8 weeks old female BALB/c ByJ mice (Charles River Laboratories), whilst for intravenous injections 5 × 106 cells were injected into the caudal vein.

Additional fourteen BALB/c ByJ mice (8 weeks old female, Charles River Laboratories) were injected in the mammary fat pad with 2500 4T1 murine breast cancer cells.

All animal experiments were approved by the “Ministère de l’Enseignement Supérieur, de la Recherche et de l’Innovation (MESRI)” (authorization numbers 2016072015478042 and 2015110618597936), and were carried out in accordance with the approved protocols.

### 2.2 MRI system

Experiments were performed on a 7*T* Bruker BioSpec system equipped with a gradient coil of 660 *mT*/*m* maximum strength and 110*μs* rise time. A volume resonator operating in quadrature mode was used for RF transmit (75.4 *mm* inner diameter, 70 *mm* active length) and a proton phased array (RAPID Biomedical GmbH) containing four elements of 30 *mm* long around an elliptic cylinder (housing: 19 × 25.5 mm^3^) was used for signal reception.

### 2.3 MRI acquisitions

A total of 55 mice were imaged after RENCA injection, 27 mice with slow-growing metastases and 28 mice with fast-growing metastases. Animals were imaged every week: from day 6 to day 32 post-implantation for fast-growing metastases mice group and from day 8 until their condition deteriorated (up to day 141 at most) for slow-growing metastases mice group. Also, the 14 mice bearing 4T1 pulmonary metastases were imaged once between day 21 and day 30 after the primary tumor implantation. Two additional healthy mice were scanned three times, two times without repositioning and one time after waking them up in order to evaluate the reproducibility of lung segmentations.

Before imaging, mice were anesthetized with isoflurane (1.5% in air) and placed in the supine position with the lungs in the center of the NMR coil. The breathing rate was monitored using an air balloon placed on top of the lungs (SA Instruments, Inc., NY). The respiration rates between mice were similar for every experiment. The 3D bSSFP images were acquired with the following parameters: TE/TR = 2/4 *ms*; flip angle (FA) = 30°; FOV: 25 × 20 × 20 *mm* ^3^; matrix: 128 × 128 × 128; resolution after reconstruction: 195 × 156 × 156 *μ* m; reception bandwidth: 100 kHZ; anterior–posterior read direction, according to Ribot et al. (2015). Four different phase offsets were used (180°; 0°; 90°; 270°) to generate four bSSFP images. Each image was acquired with four repetitions per offset. The Analog to Digital Converter (ADC) was turned on immediately after the excitation RF pulse. The corresponding acquired signal, a Free Induction Decay (FID), was recorded at each TR in addition to the echo used for imaging.

### 2.4 Image reconstruction

For image reconstruction we followed the procedure described in (Ribot et al., 2015). Briefly, the amplitude of the FID signal was characteristic of the animal’s respiration and made it possible to identify stable phases and peaks that reflected non-corrupted and motion-corrupted data, respectively. Peaks were identified using the “peak detection” function in MatLab and were then used to delete approximately 30% of the echoes distributed around these peaks. Stacks were then retrospectively reconstructed by deleting the respiration-corrupted k-space lines (or echoes) corresponding to respiration peaks. The different k-spaces obtained for each repetition were then averaged so that missing lines from one k-space could be filled by the k-space lines from another repetition. This results in a single k-space corresponding to one phase offset. All these steps were performed for each phase offset k-space to produce four complete k-spaces. FFT was then applied to generate four SG-bSSFP images containing banding artifacts. Finally, the four images obtained from the acquisition of the four different offsets were summed using the square root of the sum of square (SOS) method to produce the final SG-bSSFP image. Images were exported and processed in tiff format.

### 2.5 Image annotation

The size of acquired 3D images was 128 × 128 × 128 pixels. Each 3D image was split into 128 2D slices (see Table 1). Each 2D slice was considered as an independent image for downstream analysis. To obtain the corresponding ground truth, each slice was annotated using Fiji Schindelin et al. (2012): (i) masks were manually drawn around the lungs on every slice; (ii) masks were manually drawn around the pulmonary metastases on stacks containing metastases. Both tasks were performed by two different investigators. These annotations enabled us to select slices containing lungs or metastases. Lung and metastases masks were further concatenated with a different value for each area of interest (background, lungs, metastases).

**TABLE 1:** Summary of the dataset obtained from 186 mice. The number of annotated slices is reported alongside the final number of annotated images. Notice that the number of multiclass masks is lower than the lungs one, it is because some metastases are not annotated inside the lungs, so these slices cannot be transformed as a multiclass mask. We split this dataset into a training set and validation set with a ratio of 80/20.

—	Lungs	Metastases	Multiclass masks	Total after augmentation
Slices Number	8156	1296	5762	46096

### 2.6 Dataset

#### 2.6.1 Acquired 3D images

Three datasets have been acquired: (1) RENCA dataset with mice having two different metastases growth patterns, (2) 4T1 dataset and (3) healthy mice dataset. Image acquisitions have resulted in a total of 186 3D stacks for RENCA dataset; the 4T1 dataset contained 14 3D stacks; and the healthy dataset contained 6 separate 3D stacks.

#### 2.6.2 Annotation

RENCA dataset yielded a total of 24576 slices with 128 × 128 pixel size. Metastases were visible on 62 of the 186 3D stacks. Annotation of the RENCA dataset has resulted in a total of 8156 slices for the lungs segmentation, 1296 slices for the metastases segmentation and a total number of 5762 images with associated multiclass masks (see Table 1). Due to the imaging of the same mice at different timepoints during the development of metastases, large variation in their volume was observed, ranging from 0.0188 mm^3^ to more than 200 *mm* ^3^ (see Figure 2 for an example of slice annotation).

#### 2.6.3 Data augmentation

Notice that the resulting number of slices is quite small for training a deep neural network. Consequently, we created a data augmentation pipeline, composed of rotations and elastic transformations, which applied a factor 8 to the dataset volume (these numbers are recapitulated in the column “#Total after augmentation”, in Table 1). Rotations (90°, 180°, 270°) ensured that the network was exposed to mouse slices in every position and the elastic transformation ensured a better robustness of the network by providing a training dataset that contains slices subjected to small deformations. Together these augmentation steps helped to reduce overfitting and improved the quality of the segmentations.

#### 2.6.4 Test dataset

To create the test dataset, we selected four 3D stacks representative of the complete RENCA dataset (representing a total number of 512 slices), sampled from our dataset of 3D image stacks. It is composed of one control 3D stack without metastasis, two 3D stacks with small metastases and one 3D stack with large metastases, defined as under or above 0.4 *mm* ^3^, respectively. This test dataset was annotated by two different annotators in order to obtain a precise inter-observer variation and to quantify whether and to what degree the possible associated bias impacts the training.

### 2.7 Deep learning processing pipeline

**FIGURE 1:**
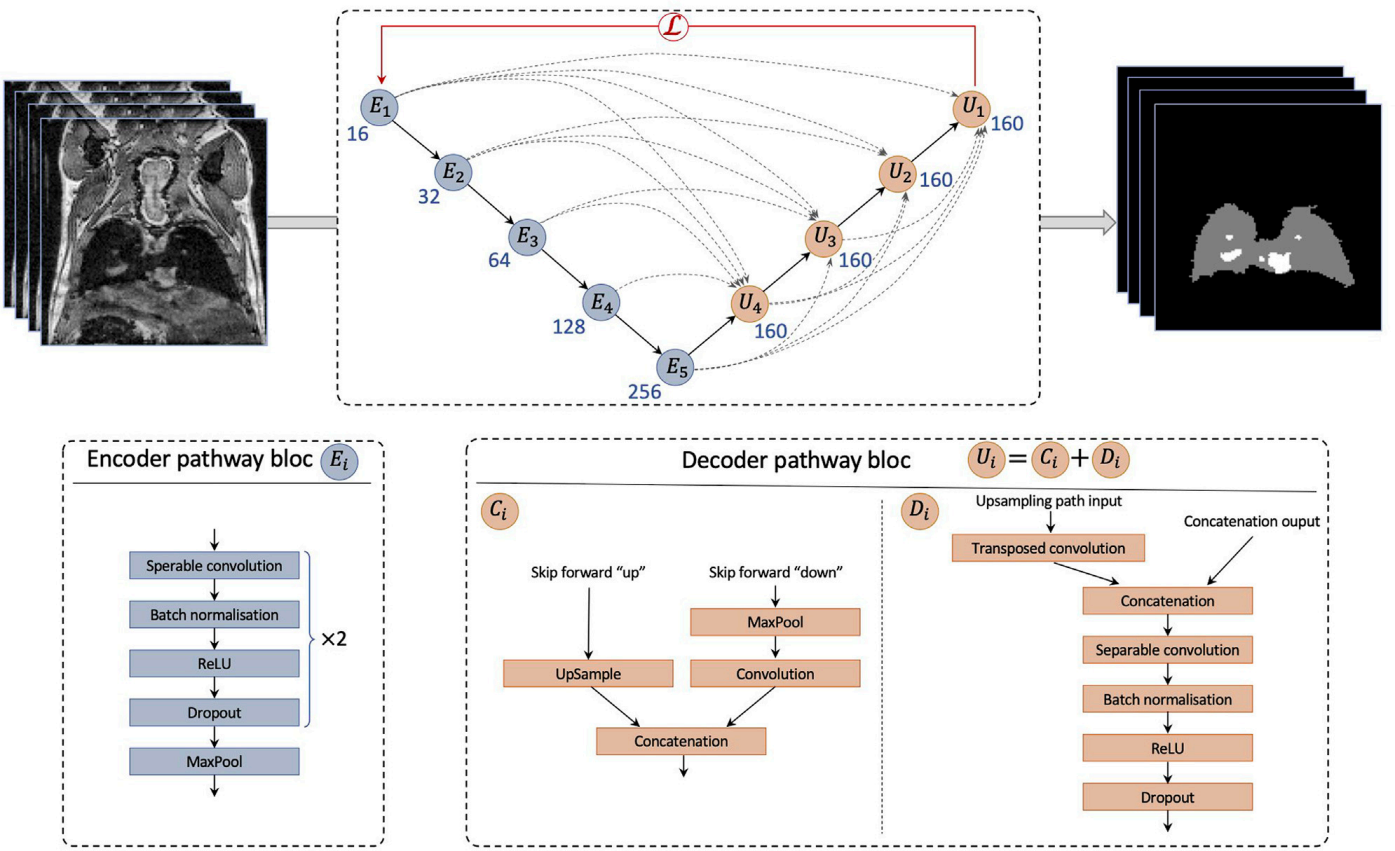
Architecture of DeepMeta network for lung and metastases segmentation. The network is composed of an encoder path and a decoder path. The encoder is composed of *E*_*i*_ blocks. The decoder is composed by *U*_*i*_ blocks composed by a concatenation block *C*_*i*_ and a decoder block *D*_*i*_. *C*_*i*_ blocks up sample or down sample feature maps into the same size as the upsampling path input and then pass it to *D*_*i*_.

DeepMeta network architecture was designed to perform multiclass segmentation and was based on the U-Net general network structure (see Figure 1). Specifically, we designed our network as a tailored implementation of the U-Net 3+ (Huang et al., 2020). The U-Net 3+ architecture uses full-scale skip connections which combine small and large feature maps from the encoder. We postulated that basing our approach on U-Net 3+ would be suitable to capture both small details and semantic features within images, and as a consequence to accurately segment both lungs and metastases. Specifically, inter- and intra-connection between the encoder *E*_*i*_ down-sampling and decoder *U*_*j*_ up-sampling pathways enabled the U-Net 3+ to account for both fine and coarse level details: in the case of DeepMeta, low level details contain the spatial and boundary information of both lungs and metastases, while high-level details encode their location.

Modifications we made to the classical U-Net 3+ architecture are the following.

• Convolution blocks of the contracting path are composed of one depthwise separable convolution (Chollet, 2017), which speeds up the training process without losing accuracy, followed by a batch norm layer, a ReLU activation and a dropout, repeated twice.
• The expanding path takes its’ usual input and concatenates it with feature maps from skip connections (*C*_*i*_) as in U-Net 3+. However, convolution blocks *D*_*i*_ are composed of a 2D transposed convolution layer, followed by the same architecture as the encoder convolutional blocks.
• The concatenated feature maps have 160 filters. The encoder starts with 16 filters for *E*_1_ and doubles this number for each consecutive block.

### 2.8 Loss

To train the network, we defined a custom loss function using a loss combination of a cross entropy, a Lovasz-Softmax loss Berman et al. (2018) and a focal loss Lin et al. (2017).

$$L=\alpha \times L_{CE}+\beta \times L_{L}+\gamma \times L_{F}$$ (1)

In our data the number of background pixels greatly exceeded that of foreground (lungs and metastases) pixels and thus one of the goals in the definition of our loss function was to solve this class imbalance. To achieve this, we chose to define $L_{CE}$ as a weighted version of the cross-entropy loss function. Indeed, the classical cross-entropy loss would have been close to 0 due to the high prevalence of True Negatives (background pixels). Moreover, for each class we defined specific weights, one for the background, five for the lungs and 15 for the metastases. The third term of the loss function $L_{F}$ , the focal loss, further tweaks the classical cross entropy for solving two issues of the classical cross entropy: the class imbalance problem and learning hard exemples. The second term $L_{L}$ , Lovasz loss, is based on the IoU and helps to obtain a better segmentation, by minimizing errors that penalize IoU the most. We have set *α* = 0.7, *β* = 0.4 and *γ* = 0.2.

### 2.9 Training

During training, the cosine annealing schedule (Loshchilov and Hutter, 2016) was used, which prevents the network from getting stuck in local minima. Experimental procedure that imaged mice at different time points allowed us to trace the metastases development but also to train the network regardless of the size of the metastasis, as we had access to a wide range of sizes. We split the slices from the training dataset using a ratio of 80% for training and 20% for validation. Network was trained on Nvidia T4 GPUs, for 100 epochs with a learning rate of 0.001 on a dedicated cluster with two two CPU Intel Xeon Silver 4114 and 128Go RAM. DeepMeta was implemented in Python 3.10 and Pytorch 1.11, for both neural network creation and training.

### 2.10 Post-processing pipeline

**FIGURE 2:**
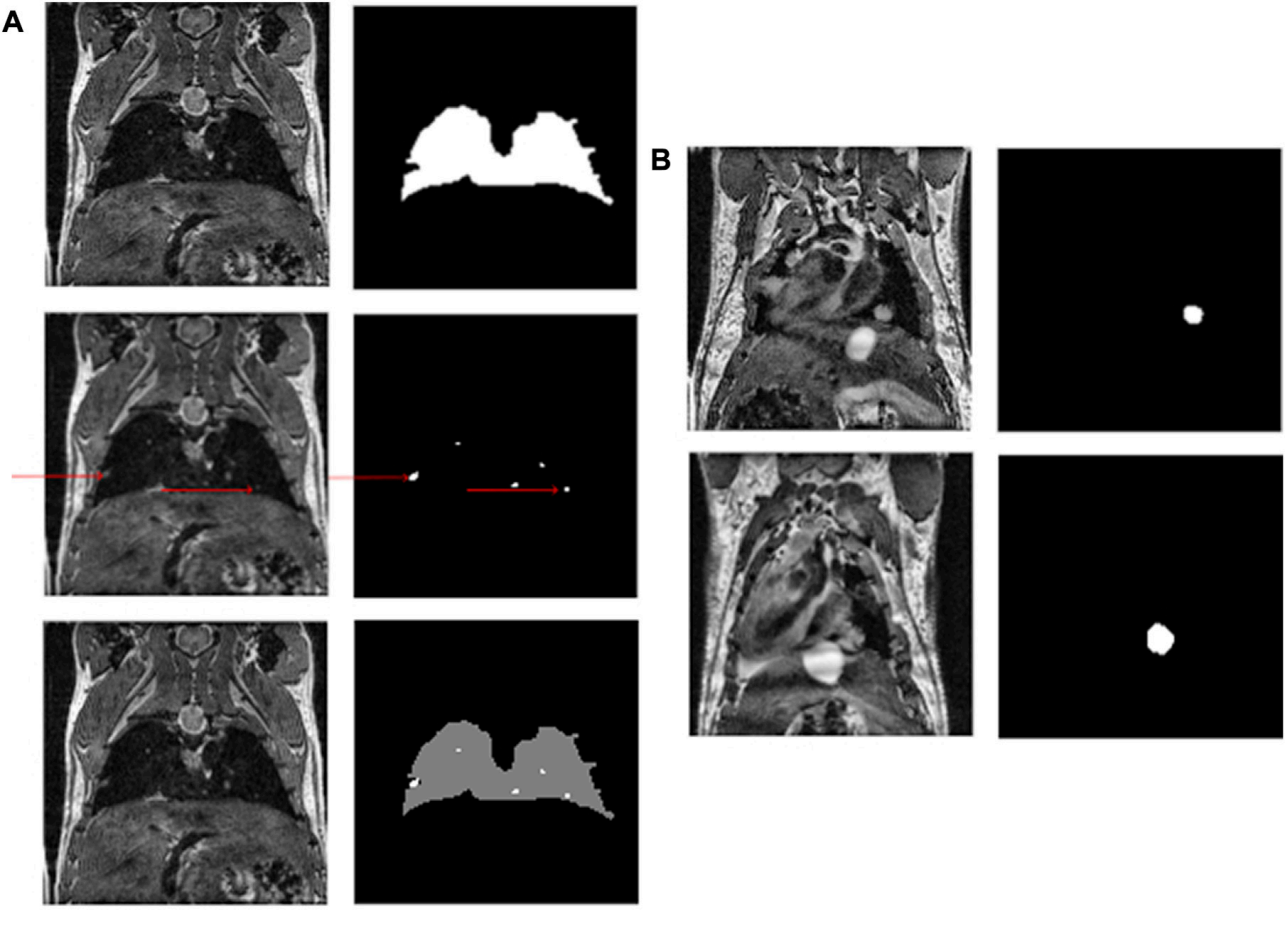
**(A)** An example of a mouse slice, the lung and metastasis masks (red arrows indicate metastases) and the multiclass resulting mask. **(B)** Shows the growth of a metastasis over time.

DeepMeta network generates segmentations in the form of masks, with values of 0 for the background, one for the lungs and two for metastases (see Figure 2). However, the output of the network required post-processing steps to improve the resulting segmentation quality. We defined a post-processing pipeline consisting of four steps.

1) The first step is to remove slices that do not contain mouse tissue. For this purpose, we applied a Laplacian of Gaussian (LoG) (SotakJr and Boyer, 1989) filter (*σ* = 7) to each slice. The Gaussian filter smoothes noise and the Laplacian operator detects edges at 0 crossing while giving a zero response in homogeneous regions. Thus, if a slice contains only noise, the Laplacian of Gaussian operator will not detect any edge and for each pixel of the slice the value yielded by the LoG filter will be close to zero. Consequently, the mean intensity of a filtered slice containing only noise will be close to zero. For this reason, we defined a threshold value of one to separate slices containing mouse tissue (⩾1) , from slices that do not contain any tissue (<1) . This processing step starts from the first slice and is stopped when the first tissues are detected. The same method is applied starting from the last slice. Once the slices that do not contain any tissue are identified, their output masks are defined to contain only 0 (background only).
2) In the second step, the mask is split into two binary masks, one mask for lungs and one mask for metastases.
3) In the third step, small blobs are removed from each of the remaining slices, more specifically blobs that are smaller than 10 pixels for lungs and three pixels for metastases (blobs are removed in the network’s output and in the ground truth in order to not bias the statistics). Additionally, a closing operation (dilation followed by an erosion) with a 3 × 3 kernel is applied to close small gaps and connect contiguous components. This results in binary masks per slice, for both the lungs and the metastases.
4) In the final step, the resulting two binary masks are concatenated to reconstitute the multiclasses mask.

Finally, to obtain each volume, we counted the number of pixels for each class in each mask of the stack and multiplied this number by the volume of one voxel, here 0.0047 *mm* ^3^, corresponding to the spatial resolution of the MR images.

In addition, to be able to compute the volume per individual metastasis (and not only the total volume), an additional step was defined. We performed the connected component analyses (Rosenfeld and Pfaltz, 1966) of the 3D stacks of metastases masks using an 18-connected neighborhood to find the instance of each metastasis and thus be able to compute its volume.

### 2.11 Evaluation

To evaluate the performance of our DeepMeta model, we used two metrics: (i) Intersection over Union (IoU), which provides a coefficient indicating how well the masks overlap, (ii) F1-score, which represents the precision and recall balance. The latter metric is particularly suited for problems with class imbalance, as it is the case in our data.

Moreover, an inter-observer comparison was performed to evaluate the dataset consistency. For a given slice three metrics were computed to compare annotations by the two experts: (1) IoU, (2) positive pixel difference and (3) a ratio of surface difference between the two masks. The IoU metric indicates how well the annotations are overlapping, i.e. whether the experts have annotated the same objects. The positive pixel difference measures the difference in mask sizes. And the ratio shows how much the surface differs in number of pixels. The ratio is calculated with the following formula: $1-\frac{\min\left(\sum_{i}p_{i},\sum_{j}p_{j}\right)}{\max\left(\sum_{i}p_{i},\sum_{j}p_{j}\right)}$ , where *p*_*i*_ are pixels from the first annotation and *p*_*j*_ are pixels from the second annotation.

## 3 Results

### 3.1 SG-bSSFP images of the mouse thorax

**FIGURE 3:**
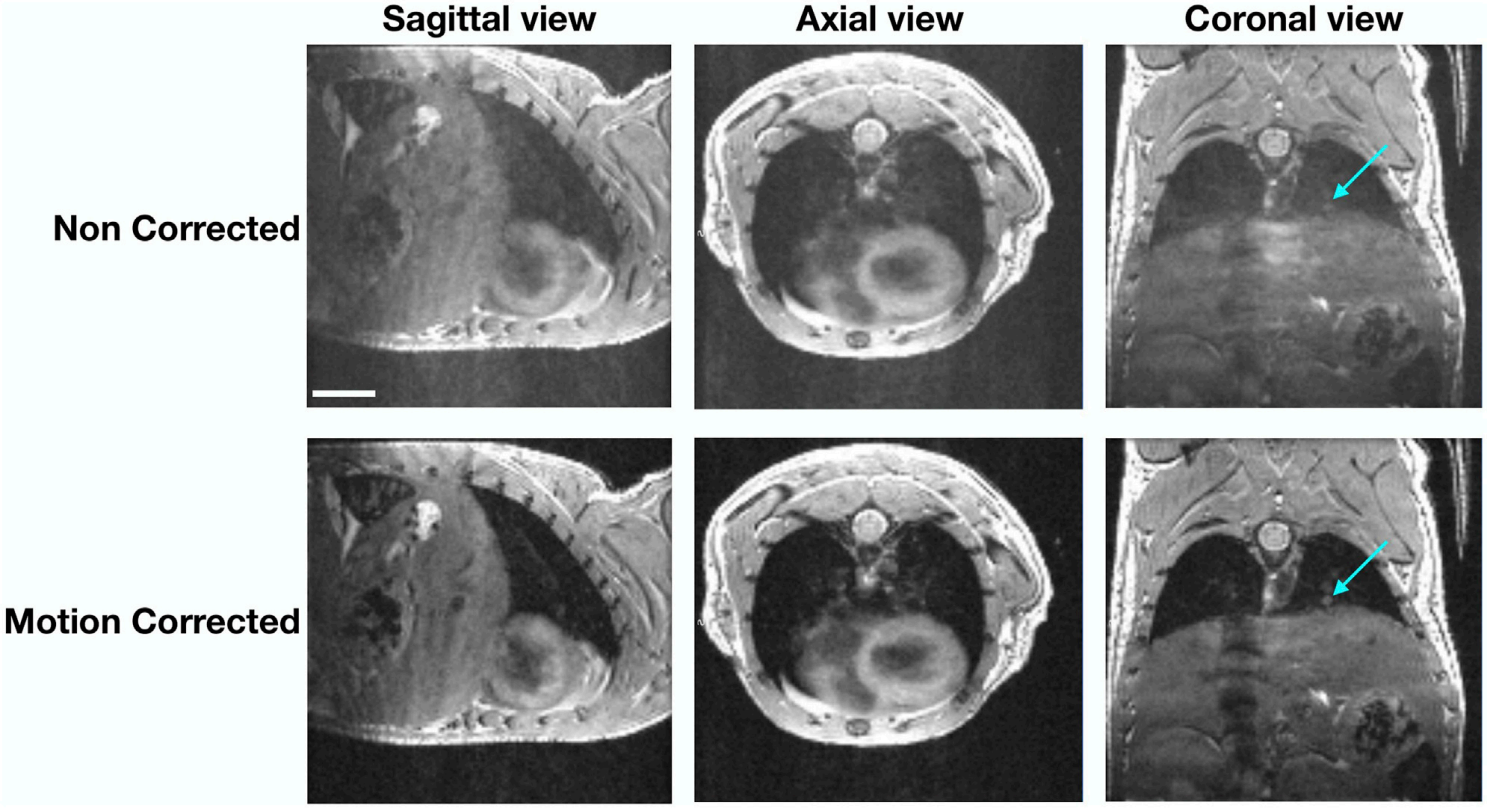
Differences between 3D bSSFP images in the 3-axis spatial direction. Images of a representative mouse before and after motion correction are shown. The arrows point to a pulmonary lesion that can be better depicted on the motion-corrected images. The scale represents 4.5 *mm*.

The SG signal could be detected even though the lungs generate low signal at this echo time (see Figure 3). The 3D SG-bSSFP images were mostly free of any motion and banding artifacts, especially in the thoracic area. As expected, lungs appeared with low signal on the images, with hyperintense areas representing blood vessels. Also, the myocardium appeared with more signal than the lungs, and the blood inside the ventricles was dark. In mice bearing pulmonary metastases, the lesions appeared in hyper-intense signals, similar to those of the blood vessels. This contrast enabled us to detect pulmonary metastases as small as four voxels, and to assess their longitudinal growth.

### 3.2 Model performance

#### 3.2.1 Assessment of our model on lungs and metastases segmentation

The performance of our DeepMeta model is shown in Table 2, considering different mice from the test set: those without metastasis (1 mouse) and those with small (2 mice) and large metastases (1 mouse). To highlight the contribution of both the U-Net 3+ architecture and of our loss function, the results are compared with both a vanilla U-Net and a vanilla U-Net 3+.

**TABLE 2:** Models performance are measured by IoU and F1-score for both lungs and metastases segmentation in our representative test set for DeepMeta, vanilla U-Net and vanilla U-Net 3+ architectures. This set includes mice without metastases and having either small or large metastases. The last column shows the mean values across the entire test set.

Object	No metastases		Small metastases		Big metastases		Total	
	IoU	F1	IoU	F1	IoU	F1	IoU	F1
*Vanilla U-Net*								
Lungs	0.86	0.93	0.86	0.88	0.76	0.84	0.84	0.88
	±0.04	±0.04	±0.07	±0.07	±0.06	±0.06	±0.05	±0.05
Metastases	NA	NA	0.69	0.74	0.70	0.64	0.70	0.69
			±0.19	±0.18	±0.17	±0.15	±0.15	±0.13
*Vanilla U-Net 3+*								
Lungs	0.87	0.91	0.86	0.90	0.80	0.87	0.84	0.89
	±0.04	±0.04	±0.07	±0.07	±0.04	±0.03	±0.05	±0.04
Metastases	NA	NA	0.71	0.75	0.62	0.67	0.68	0.71
			±0.17	±0.17	±0.16	±0.15	±0.14	±0.12
*DeepMeta*								
Lungs	0.89	0.93	0.87	0.95	0.82	0.88	0.86	0.92
	±0.03	±0.02	±0.06	±0.06	±0.05	±0.04	±0.04	±0.04
Metastases	NA	NA	0.72	0.76	0.67	0.72	0.70	0.74
			±0.19	±0.2	±0.15	±0.14	±0.14	±0.13

When interpreting these values, it should be noted that manual segmentation resulted in high inter-observer variability that could decrease to an IoU of 0.71 and 0.82 for small and large metastases, respectively. Moreover, 29% and 12% differences were measured between the two expert segmentations, highlighting the difficulty of manual metastases segmentation. Considering these constraints, the performance of the DeepMeta model for metastases segmentation is within the range of human experts.

##### 3.2.1.1 Lung segmentation

The mean volume of the mouse lungs was 463.5 *μ* L ± 72 *μ* L in the test set. The IoU index between the manual and the DeepMeta lung segmentation was 0.86 (see Table 2), improving on both vanilla U-Net and vanilla U-Net 3+. The inter-observer variability of lung segmentation was low, with a mean IoU of 0.87, leading to 8% difference (see Table 3).

**TABLE 3:** Inter-observer differences. Measured Inter-observer differences in manual annotation of the test set. Reproducibility between two annotations is measured by the IoU; annotation difference is measured by the count of differing pixels and the surface ratio, with a total pixels per slice of 16384.

Metric	Lungs	Small metastases	Large metastases
IoU	0.87	0.71	0.91
Mean difference in number of pixels	94.48	12.18	33.74
Mean surface ratio	0.08	0.29	0.12

Moreover, the reproducibility of lung segmentation was assessed on the healthy dataset that was obtained by imaging two healthy mice 3 times each. The resulting lung volume for the first mouse was 484.5 *mm* ^3^, 474.9 mm^3^ and 494 *mm* ^3^; for the second mouse: 490.4 *mm* ^3^, 512.1 *mm* ^3^ and 486.2 mm^3^. The coefficients of variance were 1.9% for the first mouse, 2.8% for the second one and 2.3% globally, showing high reproducibility for lung segmentation. The mean lung volume of the RENCA dataset is within 10% range from the mean volume of the healthy dataset, considered as reference.

##### 3.2.1.2 Metastases segmentation

The metastases were detected and automatically segmented for the test dataset. See Table 3, panel B, for an example of segmentation of a slice from the same mouse imaged at day 85 and day 92. The total metastasis volume for this mouse was 30.8 *mm* ^3^ and 47.3 *mm* ^3^ at these time points. Moreover, the model has shown high sensitivity as it could detect metastases as small as four voxels (representing here 0.0188 mm^3^) in a given slice. The IoU index of 0.72 was obtained between the manual and the DeepMeta segmentation for small metastases (see Table 2) defined as metastases with an area size of less than 85 pixels (approximately 0.4 mm^3^). This index decreased to 0.67 when metastases were larger than 85 pixels. Notice the systematic improvement relative to the vanilla architectures.

### 3.4 Capacity to distinguish different growth patterns of metastases

**FIGURE 4:**
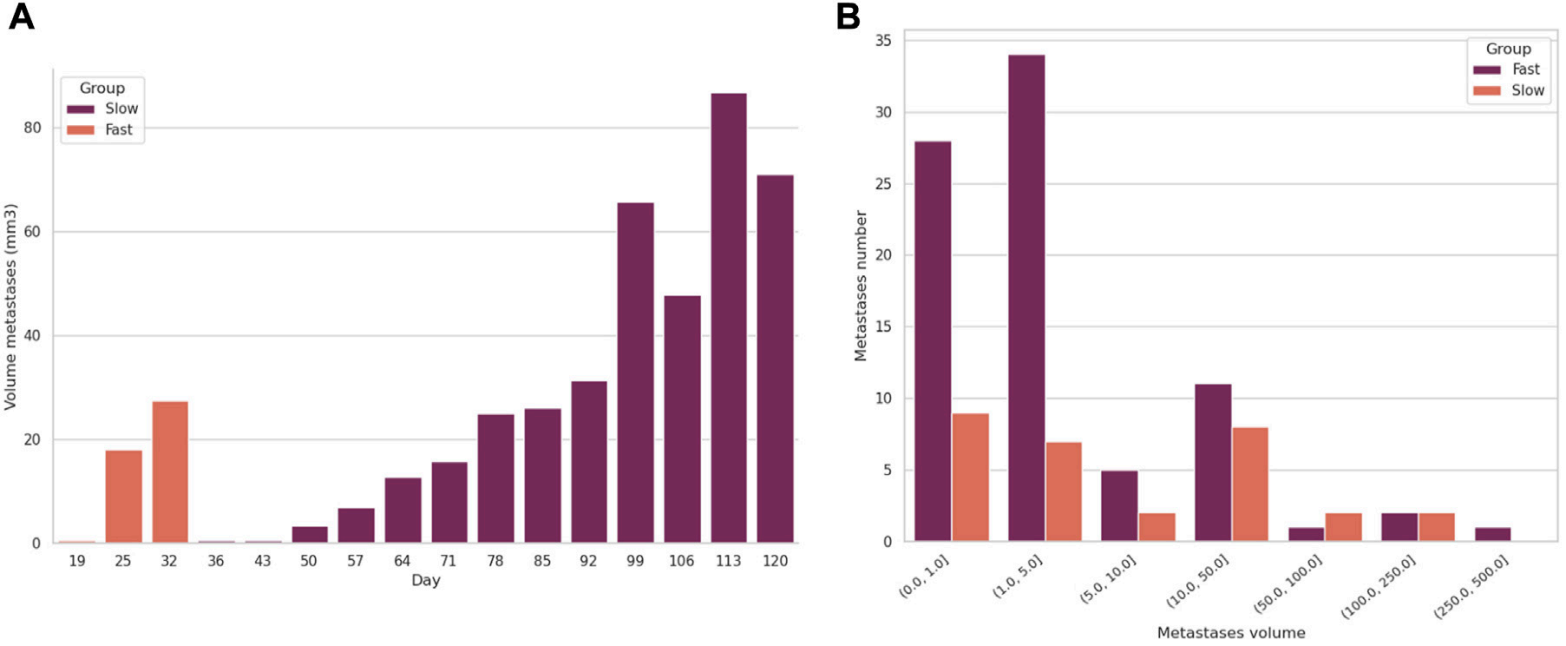
Evolution of the volume and number of metastases in two mice, a control and an il34. **(A)** shows the evolution of the total volume of metastases per mouse over time. **(B)** shows the number of metastases per volume range imaged at day 25 for control LacZ and at day 120 for il34.

In the RENCA dataset metastases were detected and segmented by our model in the fast-growing metastases mice group as early as 19 days post-injection (see Figure 4A). The metastases growing subsequently after the injection of the il34 cells became detectable at 22 days after injection. Also, the model was able to measure the lung volumes occupied by the pulmonary metastases over time. This enabled us to differentiate the two groups of mice. The DeepMeta model was also able to measure the total volume of metastases at different time points. This made it possible to count the number of metastases per volume range (see Figure 4B). Figure 4B highlights that the metastases in the fast-growing group are more homogeneous in size than the slow-growing group. Also, the slow-growing group had fewer metastases compared with the fast-growing one.

### 3.5 DeepMeta’s use case

**FIGURE 5:**
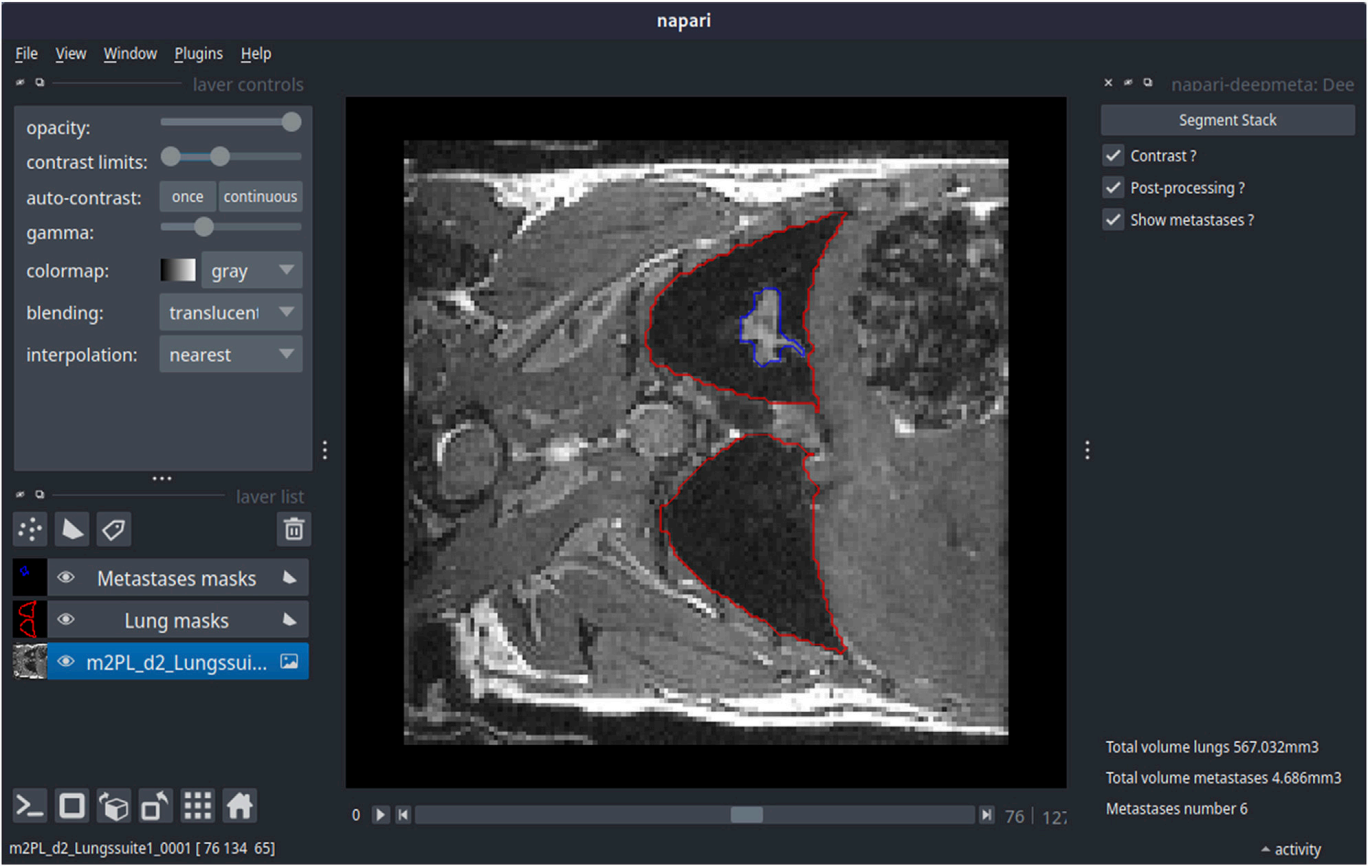
DeepMeta’s prediction on a mouse bearing 4T1 lung metastases using the deepmeta-napari plugin. On the left is the control panel of the napari interface allowing to adjust the image visualization parameters (top left) as well as the stack with contours that represent the segmentation of lungs and metastases. Lungs and metastases segmentation is shown in the central panel, in red for lungs and in blue for metastases. On the right is the DeepMeta plugin control panel. The resulting volumes for lungs and metastases are indicated on the bottom right.

To assess whether our DeepMeta model can be used to process pulmonary metastases from another cell line we applied the complete procedure from imaging to lung and metastases segmentation on the 4T1 dataset. The mean lung volume found by DeepMeta was 547 *mm* ^3^ ± 61.2 which is in the 10% range of lung volume from the healthy dataset. The network managed to also segment the pulmonary metastases (see Figure 5 and Table 4) and found an average number of 4.5 metastases per mouse with a volume range from 0.01 to 6.9 mm^3^. These results show that the DeepMeta model generalizes well to other data and in particular that it can process metastases from different cell lines. As such, this indicates that the use of DeepMeta can streamline the analysis of lungs and metastases in MR images in preclinical studies on small animals.

**TABLE 4:** Results of DeepMeta’s prediction on mice bearing 4T1 lung metastases.

Name	Lungs volume	Metastases total volume (mm)	Metastases number
4T1_1	561.3 mm^3^	1.4^3^	4
4T1_2	522.1 mm^3^	1.6^3^	15
4T1_3	586.6 mm^3^	0^3^	0
4T1_4	627.8 mm^3^	3^3^	6
4T1_5	579.8 mm^3^	0^3^	0
4T1_6	550.5 mm^3^	6.9^3^	22
4T1_7	467,5 *mm* ^3^	0.02^3^	1
4T1_8	434 *mm* ^3^	0^3^	0
4T1_9	471.4 mm^3^	0.7^3^	4
4T1_10	511.5 mm^3^	0.3^3^	4
4T1_11	599.6 mm^3^	0^3^	0
4T1_12	627.8 mm^3^	3^3^	6
4T1_13	519.8 mm^3^	0.02^3^	1
4T1_14	597.9 mm^3^	0.01^3^	1

## 4 Discussion

This study involves the optimization and development of both the image acquisition method and the processing to automatically segment mouse lungs and subsequently, the pulmonary metastases at different stages of their growth.

The MR sequence used here is the bSSFP sequence, due to its high contrasts between the lung parenchyma and pulmonary metastases and also because the 3D images can be obtained relatively fast. This sequence is commonly used for human cardiac imaging, although a 2D version is used in this case. The combination of the bSSFP sequence with a Self-Gating module makes it possible to combine high spatial resolution in the three directions and robustness to motion to detect early-growing metastases throughout the whole lung. Indeed, the bSSFP sequence can not be combined with respiration-gating in order to suppress motion artifact, so as to not perturb the steady-state of the signal. To perform lung imaging, Ultra-short Echo Time (UTE) sequences can be used to enhance the signal from the lung parenchyma. Additionally, these sequences are less sensitive to motion than Cartesian sequences because of the radial encoding. Nevertheless, due to the proton-density weighting, the contrasts between the different tissues are more homogeneous than with a bSSFP sequence, which may have decreased the detection sensitivity of the metastases and make the annotation and thus the automatic segmentation less reliable. One of the drawbacks of the radial encoding is the lower sharpness of the images. Yet, this characteristic is highly needed when measuring the volume of small structures such as pulmonary metastases.

Lungs and tumor segmentation using deep learning techniques is an active research field. In particular, CNN-based networks are popular and efficient both for 2D and 3D x-ray images (human and mouse) (Tang et al., 2019; Nishio et al., 2021; Osadebey et al., 2021; van de Worp et al., 2021). Models used for these tasks rely on a particular CNN architecture, the U-Net. As for MRI images, U-Net models are also used for brain and tissues segmentation in mice (2D and 3D) (Holbrook et al., 2020; Zhang et al., 2021). However, up to our knowledge, no model has been developed before this paper to segment lungs and metastases in small animals using MRI images.

Although conventional image processing techniques exist for lung segmentation (Egger et al., 2014), these approaches typically require human supervision to achieve high precision. Moreover, previous deep learning applications to MR images, such as En-DeepMedic (Grøvik et al., 2020), had to perform manual preprocessing steps (skull stripping before brain metastases segmentation), while DeepMeta pipeline implements what can be considered as a fully automated lung stripping and the automated metastases segmentation, both based on deep learning and automatic post-processing.

As lung slices are more susceptible to contain artifacts due to respiratory movements, we used the U-Net 3+ that helps propagate semantic and spatial information along the network reducing the network’s sensitivity to motion blur.

The U-Net 3+ architecture uses full-scale skip connection blocks that allow the network to retrieve semantic information in the reconstruction path and also prevent small objects from disappearing. In our case, lungs are not especially small objects compared to the image size (128 × 128), but metastases are relatively small and can be considered as small objects. A previous study (Zhang et al., 2021) shows that classical architectures do not perform well on small objects. Thus, this U-Net 3+ based network architecture is particularly suited for the task of small object detection.

DeepMeta U-Net 3+ model enables the reconstruction of lung volumes consistent with previous studies (Heverhagen et al., 2004; Soutiere and Mitzner, 2004; Ribot et al., 2011), thus ensuring the viability of this approach. Note that the chosen supervised learning approach relies on manual data annotation for both lungs and metastases. For example, lung segmentation alone was performed on 8156 slices, which is both time consuming and potentially biais-prone. A possible avenue to augment the dataset with known ground truth could be by data augmentation based on function decomposition from a template (Tustison et al., 2019). This technique is promising for lung data augmentation, but not for metastases. Indeed, these lesions have their shape and volume that grow over time, and their location varies among mice.

The high variability between annotators, especially for metastases, might come from partial volume effects that can greatly modify the volume of small metastases. For larger metastases, especially those growing close to the heart or the thoracic muscles, the main issue comes from the proper delineation of the metastases, as all these structures show similar signals on the bSSFP images.

The method presented here is the first stone to further developments to make deep learning models more robust to variability. First, imaging with different reception coils would enrich the training dataset. Indeed, our study was conducted using a prototype coil that is not widely used in imaging laboratories. It will thus be relevant to train the DeepMeta models with images generated by more commonly used coils, such as surface or volume coils. In addition, different models of pulmonary metastases could be evaluated. Indeed, their shape and growth pattern usually depend on the cancer cells from which they originate. Some metastases grow along blood vessels (co-option), while others develop neo-angiogenesis; the RENCA cells used here grow like spheres, whereas others have a very invasive phenotype, *etc.*

### 4.1 Limitations

Deep learning networks do not carry object semantics, which results in the network segmenting any object that has a similar structure to the metastases (e.g., blood vessels). The performance of the network depends on the architecture and on the training and thus on the training dataset. In this work, the images were acquired using the bSSFP MRI sequence, which implies that the pre-trained DeepMeta models are expected to perform well on similarly acquired images, but that the performance might decrease if the images to be segmented are acquired with a different sequence. Consequently, retraining might be necessary by including images acquired with other MRI sequences in the training dataset to achieve a higher level of generalization.

The bSSFP sequence has several advantages for mice imaging. Nevertheless, the inherent banding artifacts severely affect the quality of the images. It is thus necessary to acquire images at multiple phase offsets, which lengthens the acquisition time. Our MR reconstruction pipeline also required manual selection of the most sensitive coil to the motion self-gated signal variation and the rejecting window size. Nevertheless, the whole reconstruction pipeline can be fully automated through the use of an advanced signal processing pipeline such as SSA-FARY (Rosenzweig et al., 2020).

In conclusion, we have developed a freely available and highly reproducible deep learning model that allows segmentation of lungs and metastases as well as the measurement of the corresponding volumes over time in small animal MR images without need for human intervention.

## Data Availability

The datasets presented in this study can be found in online repositories. The names of the repository/repositories and accession number(s) can be found below: https://zenodo.org/record/7014776.
